# Metabolic profiling during *ex vivo* machine perfusion of the human liver

**DOI:** 10.1038/srep22415

**Published:** 2016-03-03

**Authors:** Bote G. Bruinsma, Gautham V. Sridharan, Pepijn D. Weeder, James H. Avruch, Nima Saeidi, Sinan Özer, Sharon Geerts, Robert J. Porte, Michal Heger, Thomas M. van Gulik, Paulo N. Martins, James F. Markmann, Heidi Yeh, Korkut Uygun

**Affiliations:** 1 Center for Engineering in Medicine, Dept. of Surgery, Massachusetts General Hospital, Harvard Medical School, Boston, MA, USA; 2 Department of Experimental Surgery, Academic Medical Center, University of Amsterdam, Amsterdam, The Netherlands; 3 Section of Hepatobiliary Surgery and Liver Transplantation, Department of Surgery, University Medical Center Groningen, University of Groningen, Groningen, The Netherlands; 4 Transplant Center, Dept. of Surgery, Massachusetts General Hospital, Boston, MA, USA; 5 Transplant Division, Dept. of Surgery, University of Massachusetts, Worcester, MA, USA

## Abstract

As donor organ shortages persist, functional machine perfusion is under investigation to improve preservation of the donor liver. The transplantation of donation after circulatory death (DCD) livers is limited by poor outcomes, but its application may be expanded by *ex vivo* repair and assessment of the organ before transplantation. Here we employed subnormothermic (21 °C) machine perfusion of discarded human livers combined with metabolomics to gain insight into metabolic recovery during machine perfusion. Improvements in energetic cofactors and redox shifts were observed, as well as reversal of ischemia-induced alterations in selected pathways, including lactate metabolism and increased TCA cycle intermediates. We next evaluated whether DCD livers with steatotic and severe ischemic injury could be discriminated from ‘transplantable’ DCD livers. Metabolomic profiling was able to cluster livers with similar metabolic patterns based on the degree of injury. Moreover, perfusion parameters combined with differences in metabolic factors suggest variable mechanisms that result in poor energy recovery in injured livers. We conclude that machine perfusion combined with metabolomics has significant potential as a clinical instrument for the assessment of preserved livers.

Liver transplantation is currently the only curative treatment option for patients suffering from end-stage liver disease. However, transplantation continues to be limited by organ availability. A great number of donor livers are deemed unsuitable for transplantation and are discarded. The suitability of organs for transplantation is determined on the basis of empirically-established clinical parameters that have been shown to result in increased rates of early allograft dysfunction or biliary complications. Such clinical parameters, including prolonged warm ischemia (>30 min) during donation after circulatory death (DCD)^1^ and hepatosteatosis^2^, are related to an increased susceptibility of the organ to ischemia and reperfusion (I/R) injury during liver preservation and transplantation. Alleviation of I/R injury as well as improved viability testing before the transplantation are likely to extend the utilization of otherwise suboptimal organs.

To this end, functional *ex vivo* machine perfusion is gaining clinical interest for the recovery and assessment of liver grafts prior to transplantation. A paradigm-shift from metabolic/functional suppression during cold storage to metabolic support in (sub)normothermic liver perfusion potentiates a more active recovery and more representative evaluation of the metabolic state before transplantation. Various pre-clinical models have shown improved transplant outcome following functional machine perfusion at both normothermic (body temperature; ±37 °C) and subnormothermic temperatures (±21 °C)^3^,^4^, maintaining or even improving function in initially discarded human grafts^5^,^6^. The feasibility of perfusion preservation as an alternative to static cold preservation is supported by initial clinical results with hypothermic machine perfusion^7^,^8^.

Importantly, *ex vivo* assessment of organ quality prior to implantation could allow dynamic assessment of recovery during perfusion by providing an accurate indication of whether a liver has been sufficiently improved. This approach may also replace the currently employed appraisal of liver viability with in-depth profiling of hepatic function on a liver-to-liver basis. Although several *ex vivo* viability indicators such as bile production^9^, adenosine triphosphate content^6^, and hepatic aminotransferase release^10^ are used during functional liver perfusion, very little is known about the molecular mechanisms of *ex vivo* recovery of the liver and the metabolic processes that underlie functional recovery.

Innovative metabolomic analysis techniques have enabled the identification of key pathways and mechanisms involved in hepatobiliary disease and have revealed biomarkers associated with donor liver function^11^,^12^,^13^. Since metabolomics involves the study of downstream products of multiple physiological and pathological processes, it may offer a unique broad-spectrum perspective of the transplant liver – an organ that hosts an unequaled plethora of metabolic interactions^14^. The rapid change in the metabolic conditions of to-be-transplanted livers during the perioperative period warrant metabolomic assessment. However, while metabolic recovery plays a vital role in clinically successful machine perfusion, the metabolomic signatures of *ex vivo* perfused livers remain underexplored.

To address this, we performed a comprehensive assessment of human livers in a functional subnormothermic machine perfusion (SNMP) platform (Fig. 1). Moreover, groups of steatotic DCD livers (warm ischemia time (WIT) <30 min) and livers with severe warm ischemia (>30 min) were compared to control DCD livers (WIT <30 min), which met transplantation criteria, to assess the potential of metabolic profiling in discriminating between various states of injury.

## Materials and Methods

### Study protocol

Twenty-one human livers were evaluated during 3 hours of SNMP. Livers were procured in the New England Organ Bank (NEOB) region, following standard procurement technique for orthotopic transplantation, and were rejected for transplantation by all offered transplant centers and consented for research. Livers were donated after circulatory death (DCD; n = 18) or after brain death (DBD; n = 3). To maximally standardize the experimental approach and reduce confounding variables in human liver donors and the procurement surgery, metabolomic analysis was performed on DCD livers that met the following criteria. Nonsteatotic (0% macro- and microvesicular) livers that met DCD transplantation criteria (including WIT <30 min) and were discarded for logistical reasons were used as DCD controls (DCD <30 min group (WIT range 19–23 min); min *n* = 3). Nonsteatotic DCD livers with severe warm ischemia (DCD >30 min group (WIT range 36–54 min); *n* = 3) and DCD livers discarded with moderate-severe (>33% macro/micro-) steatosis and WIT of <30 min were analyzed (Steatotic DCD group; (WIT range 16–27 min); *n* = 3) as groups with severe injury. All livers were from donors less than 70 years of age (Table S1).

Wedge liver biopsies (300–500 mg) were taken before, and hourly during SNMP and used for histological-, ultrastructural-, metabolomic-, and biochemical analysis. Samples of the perfusate were collected every 30 min and were assessed for hepatic injury and function markers. As controls, liver samples (*n* = 12) were collected from patients undergoing bariatric surgery for weight loss indications, where the WIT was kept to a minimum (<5 min). Informed consent was obtained from the patients before surgery. These samples varied in fat content from 0–5% (*n* = 6), 5–66% (*n* = 4) to >66% (*n* = 2), successfully representing the variation in perfused livers. All biopsies underwent frozen homogenization.

The Massachusetts General Hospital Institutional Review Board (IRB) and the New England Organ Bank (NEOB) approved this study (No. 2011P001496) and all studies were carried out in accordance with IRB and NEOB approved guidelines.

### Subnormothermic machine perfusion platform

Validation and technical details of the system for support of human livers can be found elsewhere^6^. Briefly, the SNMP system provided oxygenated (95% O_2_, 5% CO_2_) perfusion of the hepatic portal vein (PV) and artery (HA) with a nutrient-rich, cell-free, and oxygenated perfusate. Organ temperature was ambiently maintained at 21 °C.

### Determination of liver function

Oxygen uptake was calculated from the difference between in- and outflow dissolved oxygen content as described elsewhere^6^. Liver function was determined using the indocyanine green (ICG) clearance test. ICG clearance was measured in seven livers at random (indicated in Table S1). At the end of perfusion, 10 mg ICG was added to and thoroughly mixed in the organ bowl and left to circulate with the perfusate for 3 minutes. Samples were collected every 2 minutes for 25 minutes and a final sample after 30 minutes. The concentration of ICG in the perfusate was measured spectrophotometrically at 800 nm.

### Biochemical assays

*ATP assay*. Absolute levels of adenosine triphosphate (ATP) were determined in liver biopsies with a luminescence-based assay (Cell Viability Kit; Biovision). Briefly, nucleotides were extracted from ~1 mg of pulverized tissue and peak luminescence was measured for 60 seconds in a single tube. ATP concentrations were normalized to tissue protein content determined by the BCA protein assay (Thermo Fisher Scientific, Waltham, MA).

*Alanine transaminase assay.*  ALT concentration was determined in the perfusate by assessing the enzymatic conversion of L-alanine by ALT and LDH in a kinetic assay (Infinity Reagent; Thermo Fisher Scientific).

### Targeted metabolomics – cofactor analysis

Crushed tissue biopsies (~25 mg) from the three aforementioned groups were analyzed for metabolic cofactors (ATP/ADP/AMP, NADH/NAD^+^, NADPH/NADP, FAD, and GSH/GSSG) using targeted multiple reaction monitoring analysis on a 3200 QTRAP LC/MS-MS (Triple quadrupole liquid chromatography – mass spectrometry) system (AB Sciex, Foster City, CA). For a more detailed description of the methodology readers are referred to the supplementary methods.

### Untargeted metabolomics analysis

Untargeted profiling of primary metabolites was performed using GC-TOF-MS (gas chromatography–time of flight–mass spectrometry). This analysis detected 159 metabolites and was run for the three aforementioned groups at 2 time points (t = 0 and t = 3 h), each with 3 replicates. Detailed methodology is described in the supplementary methods.

### Histological and ultrastructural assessment

*Light microscopy.* Liver biopsies were fixed in 10% buffered formalin and transferred to 70% ethyl alcohol for further histological processing. Paraffin-embedded samples were sectioned and stained with hematoxylin and eosin (H&E). The grade of both micro- and macrovesicular steatosis was determined by light microscopic assessment of H&E slides grade 0, 0%; grade 1, <10%; grade 2; 10–33%; grade 3, 33–66% and grade 4, >66%.

*Transmission electron microscopy (TEM).*  Multiple ~1 mm^3^ liver sections were fixed in EM grade, 2.5% glutaraldehyde, 2.5% formaldehyde and 0.1 M sodium cacodylate buffer (pH = 7.2), washed twice in 0.1 M sodium cacodylate buffer and fixed with 1.0% osmium tetroxide. Fixed samples were alcohol dehydrated with propylene oxide and embedded in Epon. Sixty-nanometer sections were cut using a diamond knife ultramicrotome (LKB Ultramicrotome, Bromma, Sweden) and viewed and imaged with a Philips 410 transmission electron microscope (Philips Electronics, Eindhoven, the Netherlands). Transmission electron microscopy was performed at the Northeastern University Center for Electron Microscopy^15^.

Mitochondrial scoring was adapted from Ben Mosbah *et al.*^16^. Mitochondria in a minimum of 20 hepatocytes from a minimum of 3 sections per liver were scored as follows: 0, normal mitochondria; score 1, prominent cristae; score 2, swelling; presence of electron-dense amorphous material.

### Statistical analysis and data processing

Statistical analysis was performed in GraphPad Prism (GraphPad Software, San Diego, CA). Differences in cofactor and metabolite levels between pre- and post-perfusion were analyzed by Wilcoxon-signed rank test or paired student’s *t-*test following normality testing (Kolmogorov-Smirnov test), or repeated measures analysis of variance (ANOVA) when comparing 3 time points. Statistical analysis of normally distributed data sets was performed with an unpaired student’s *t*-test. Data are expressed as median ± interquartile range, unless otherwise specified.

Methods for principal component analysis and heat map construction are provided in the supplementary methods.

## Results

### Liver function and hepatocellular injury during SNMP

**Figure 2:**
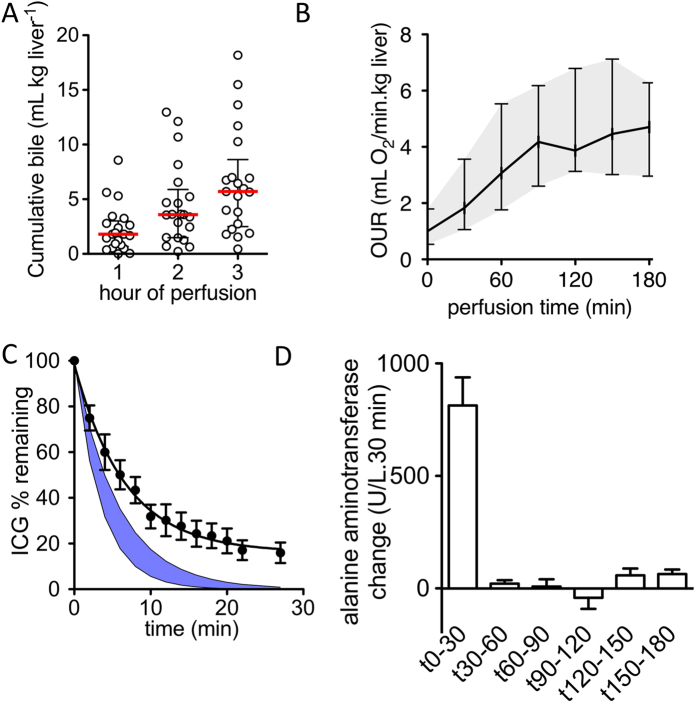
Characterization of function and injury. (**A**) Cumulative production of bile. Bile flow was measured hourly and was sustained for 3 hours of SNMP (n = 21, mean ± sem) (**B**) Oxygen uptake from the perfusate. The oxygen uptake rate calculated from in- and outflow partial oxygen (*p*O_2_) increased substantially during the first two hours of SNMP preservation (n = 21, mean ± sem (shaded)) (**C**) Clearance of indocyanine green (ICG). The postperfusion clearance of ICG was reduced from reference values (blue shaded) under *ex vivo* subnormothermic conditions (n = 7, mean ± sem (shaded)). (**D**) Release of alanine aminotransferase. Significant ALT release was observed in the first 30 minutes, after which release of ALT was reduced to a minimum for the remainder of the SNMP (n = 21).

As an initial assessment, various aspects of hepatic function were evaluated during SNMP of discarded DCD and DBD livers (*n* = 21). A sustained production of bile was observed during SNMP, ranging from 0.45–18.2 mL • kg^−1^ liver/3 hours (Fig. 2A). Oxygen uptake, estimated from the difference between in- and outflow oxygen content, increased from 1.19 to 5.15 mL O_2_ • min^−1^ • kg^−1^ liver in the first 2 hours (P < 0.001), after which it plateaued (Fig. 2B).

To assess hepatocellular uptake and clearance function, ICG was administered after 3 hours of perfusion and clearance from the perfusate was monitored for an additional 30 minutes. A perfusate clearance rate of 15.0 ± 2.4% • min^−1^ (95% CI) was observed (Fig. 2C), just below the normal range of plasma disappearance rate (16–25% • min^−1^)^17^. SNMP did not induce hepatocellular injury, as no increase in alanine aminotransferase (ALT) was observed after the initial washout phase (Fig. 2D).

### Cofactor and energy status in the liver during subnormothermic machine perfusion

**Figure 3:**
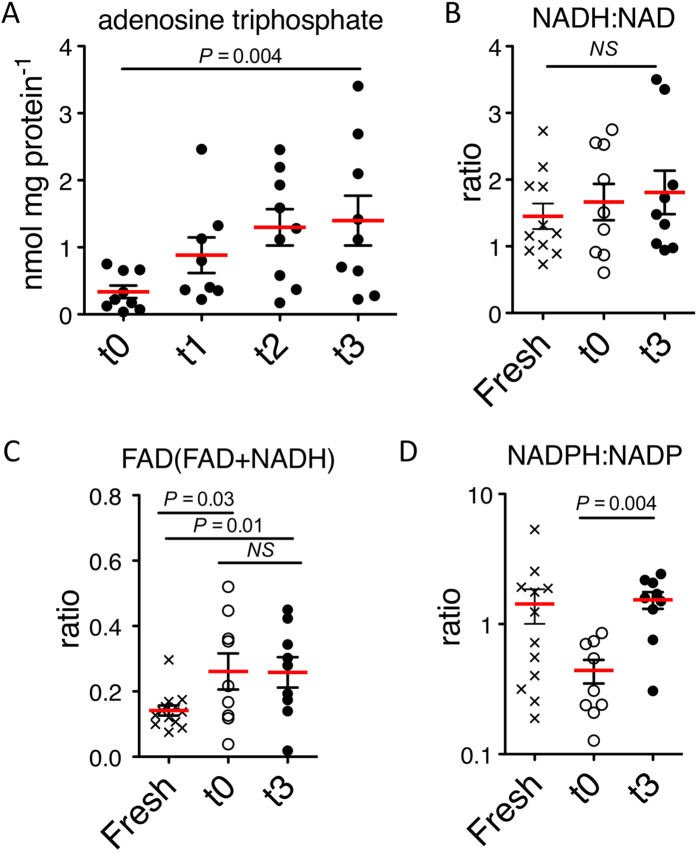
Analysis of adenosine and redox cofactors in human liver tissue. (**A**) ATP concentration in biopsies from SNMP- preserved liver grafts. Absolute ATP content increased significantly during SNMP preservation (n = 9, *P *=* *0.03) (**B**) NADH:NAD ratios in human liver biopsies before (n = 9) and after (n = 9) SNMP compared to fresh samples (n = 12). The ratio NADH:NAD was unchanged during SNMP (n = 9) and did not significantly differ from fresh samples (n = 12) at either time point (*P* ≥ 0.05) (**C**) Estimated FAD were increased after ischemia, relative to fresh samples (*P* = 0.03) and remained unchanged during perfusion (n = 9, *P* ≥ 0.05) (**D**) NADPH:NADP ratios increased significantly during SNMP (n = 9), and were restored to fresh liver levels (n = 12; *P *=* *0.0012). Bars represent mean ± sem.

Metabolic cofactors were measured in the aforementioned 3 groups (n = 9 discarded livers) and in intraoperative control samples (n = 12 liver biopsies). A significant increase in ATP content was observed after 3 hours of SNMP (4.12-fold, *P* = 0.004) (Fig. 3A), as well as a not statistically significant increase in the ratio of ATP to ADP (2.9-fold, *P* = 0.07) and energy charge (defined as: (ATP + 1/2ADP)/(ATP + ADP + AMP)) (1.93-fold, *P* = 0.12), which did not reach significance (Fig. S1A/B). Compared to biopsies taken from fresh livers, the ATP:ADP ratio as well as the energy charge decreased slightly, yet this did not reach statistical significance (*P* < 0.1).

The ratio of flavin and nicotinamide is the result of NAD^+^/FAD reduction pathways and oxidation by the electron transport chain, driving oxidative phosphorylation. The ratio NADH:NAD^+^ was not significantly altered by perfusion, and both pre- and postperfusion measurements were not significantly different from fresh samples (Fig. 3B). The reduced form of FAD (FADH_2_) was not detectable by this method, but flavin cofactor ratios could be approximated by the change in FAD:(FAD + NADH), which were similarly unchanged during perfusion (*P* > 0.05; Fig. 3C). Fresh values of FAD:(FAD + NADH) were significantly lower than both pre- and postperfusion levels (*P* ≤ 0.03). Relative to fresh liver samples, a significant decrease in NADPH:NADP^ + ^was observed preperfusion, which returned to fresh levels after SNMP (Fig. 3D; 3.48-fold, P = 0.004).

### Metabolomic changes in the liver during subnormothermic machine perfusion

**Figure 4:**
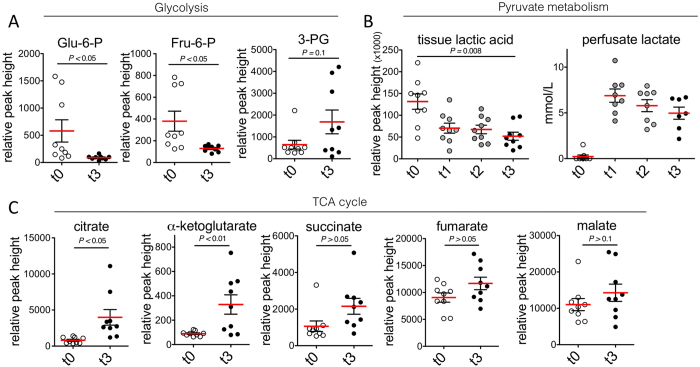
Metabolite changes of key metabolic pathways. (**A**) Relative values of detected glycolytic intermediates; Glu-6-P, glucose-6-phosphate, Fru-6-P, fructose-6-phosphate; 3-PG, 3-phospoglycerate. Glu-6-P and Fru-6-P decreased significantly during perfusion (*P *=* *0.03*, P *=* *0.015, respectively), while 3-PG showed an insignificant increase (*P* = 0.09) (**B**) Relative values of lactic acid in tissue and perfusate concentration of lactate. A large release of lactate was observed in the first hour, after which both tissue lactic acid and perfusate lactate levels decreased significantly (*P *=* *0.004). (**C**) Relative values of intermediates of the TCA cycle. Significant increases are observed in levels of citrate, α-ketoglutarate and succinate (*P* < 0.05). Changes in these metabolic pathways represent a reversal of ischemic metabolic shifts and suggest an increase in TCA cycle activity. Bars represent mean ± sem.

An untargeted metabolomics analysis was performed on machine perfused hepatic tissue of the three aforementioned groups to determine levels of metabolites relevant to ischemia and energy metabolism. The relative concentrations of 159 annotated primary metabolites were determined. Table 1 summarizes all near-significantly (*P* < 0.10) changed metabolites during perfusion (t0–t3), stratified according to metabolite class and associated pathways derived from the Human Metabolome Database (HMDB)^18^ and the Kyoto Encyclopedia of Genes and Genomes (KEGG)^19^. Remarkable statistically significant (*P *<* *0.05), or nearing significant (0.10 ≥ *P* ≥ 0.05) changes amongst primary metabolites are seen in sugar compounds, amino acids and derivatives, as well as carboxylic-/keto- and hydroxyl acids (Table 1). Glucose-6-posphate and fructose-6-phosphate were significantly decreased (*P* < 0.05), while 3-phosphoglycerate did not increase significantly (*P* = 0.10) (Fig. 4A). In the first hour of SNMP, accumulated tissue lactic acid is released into the perfusate as lactate. After the first hour, a decrease in both tissue and perfusate lactate content was found, reflecting oxidation back to pyruvate (*P* = 0.008) (Fig. 4B). Alterations were found in TCA cycle intermediates that were most proximal to pyruvate, with significant increases in citrate (4.95-fold; *P* < 0.05) and α-ketoglutarate (3.86-fold; *P* < 0.01) (Fig. 4C). Differences in TCA metabolite levels between the t0 and t3 time points tapered for the metabolites more downstream in the TCA cycle (i.e., from succinate onward).

### Metabolomic phenotypes of severely ischemic and steatotic injury during SNMP

In a subsequent analysis, liver groups with differing degrees of injury and parenchymal hepatopathology were compared. As a control group, DCD livers with a WIT of <30 min were used (*DCD *<* 30* min group). DCD livers with a long WIT of >30 min and steatotic livers (macrosteatosis >33%; WIT of <30 min) were used as severe injury groups (*DCD > 30* min group and *Steatotic DCD* group).

**Figure 5:**
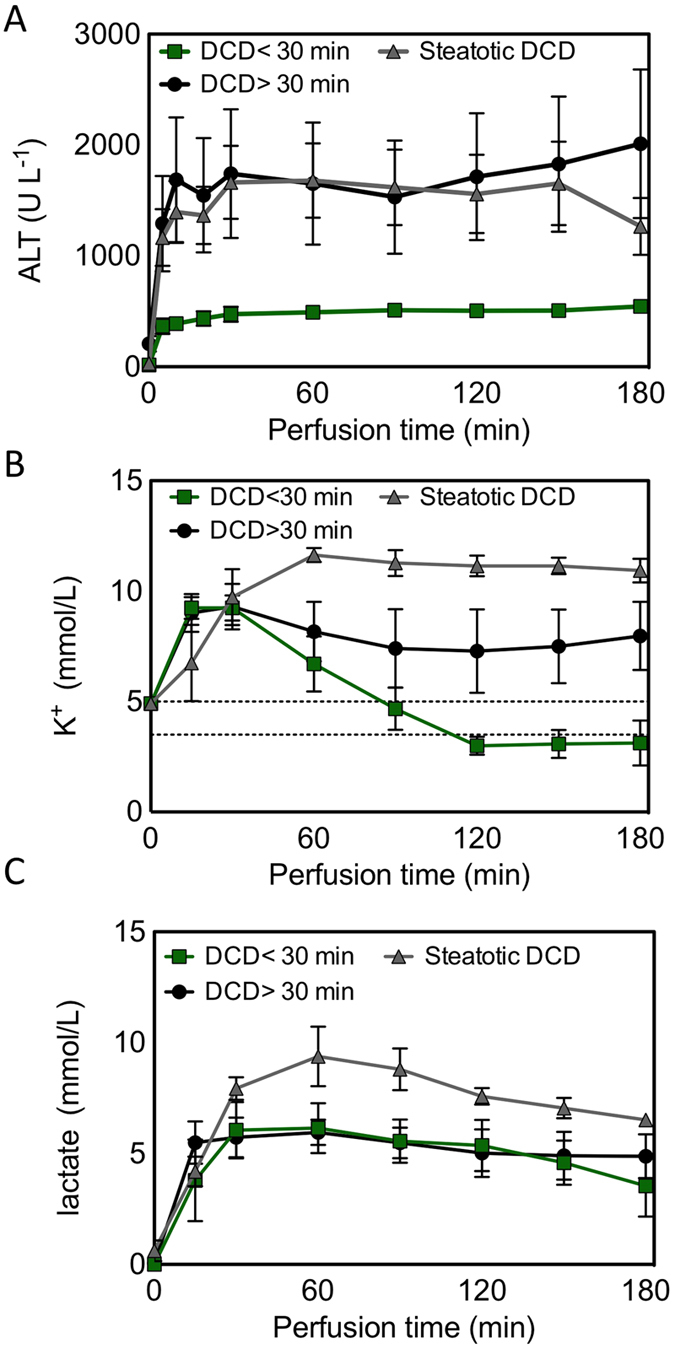
Injury markers between control DCD, steatotic, severely warm ischemic livers. (**A**) Levels of alanine transaminase (ALT), (**B**) potassium, and (**C**) lactate in the perfusate throughout 3 hours of SNMP. Dashed lines reflect normal blood reference values for potassium. Data shown as mean ± sem.

**Figure 6:**
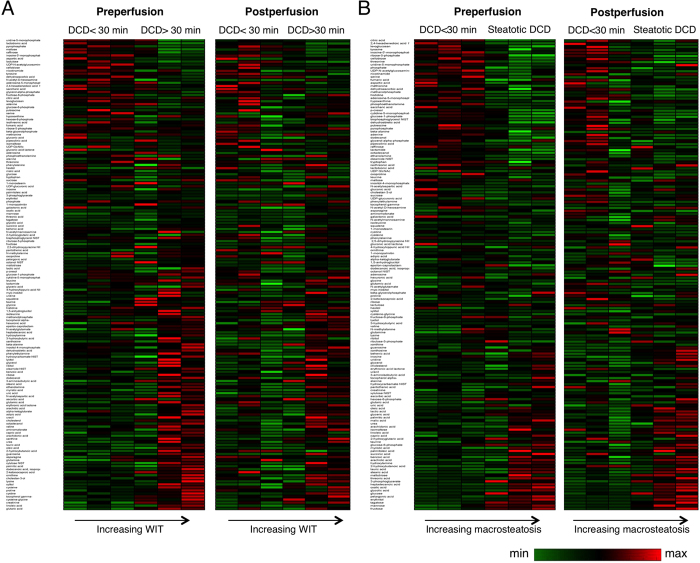
Metabolomic profile of ischemic and steatotic injury. (**A**) Heatmap visualization of untargeted metabolomic assessment of preperfusion and postperfusion liver grafts. DCD livers with a WIT <30 min (DCD <30 min) are compared to DCD livers with severe warm ischemia (DCD >30 min). The heatmap on the left shows the profile preperfusion and the right shows the profile postperfusion. (**B**) DCD <30 min livers are compared to steatotic DCD livers (right). From left to right, the macrosteatosis scores are 0, 0, 0, 2, 3, 3. The heatmap on the left shows the profile preperfusion and the right shows the profile postperfusion. Individual livers are arranged in columns with increasing warm ischemia from left to right, metabolites are ordered by slope correlating relative metabolite levels to ischemia or steatosis. The color gradient used to color the entries ranges from green to red, corresponding to relatively low and high abundance of the metabolite respectively. Both DCD >30 min and steatotic DCD livers could be discriminated from DCD <30 min livers based on their visual metabolomic pattern.

Compared to the DCD <30 min group, more extensive injury was observed in the severe injury livers, as evidenced by the increased alanine aminotransferase (ALT) (*P* = 0.03), potassium (*P *=* *0.01), and lactate levels (steatotic DCD livers only; *P *=* *0.0002) in the perfusate (Fig. 5). Common effects of SNMP notwithstanding, we anticipated that livers with varying degrees of warm ischemic and steatotic injury would reveal distinguishing metabolomic profiles. A heat map representation in Fig. 6 shows relative concentrations of primary metabolites before and after perfusion. Nonsteatotic livers showed a clear change with increasing warm ischemia time, both before and after perfusion (Fig. 6A). Similarly, steatotic DCD livers could be discriminated from the DCD <30 min group based on their metabolic profile (Fig. 6B). Metabolites that showed a significant or near-significant correlation between WIT and steatosis before and after SNMP are listed in Table S2.

**Figure 7:**
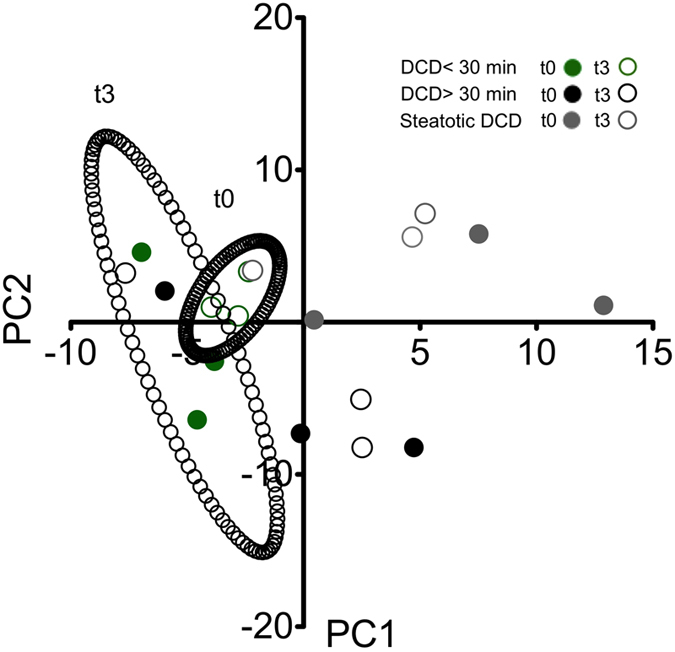
Multivariate analysis of pre- and postperfusion metabolomic profile. Principal component analysis of DCD <30 min (green), DCD >30 min (black), and steatotic DCD livers (grey) pre- (open circles) and postperfusion (filled circles), providing a 2-dimensional (PC1/PC2) representation of the untargeted metabolomic profile. Clustering of control livers is seen, both pre- and postperfusion. All but one of the steatotic DCD and DCD >30 min livers show separation from the DCD <30 min livers, lying outside the 95% confidence interval of the pre- and postperfusion controls (black ellipses).

Principal component analysis of the 159 primary metabolites was performed and included the pre- and postperfusion profile, which showed clustering of DCD <30 min group (Fig. 7). Remarkably, one liver in both the steatotic DCD and the DCD >30 min group was identified that appeared to more closely resemble the DCD <30 min group in the PCA. Interestingly, these livers were the least steatotic (macrosteatosis score 2) and had the shortest duration of warm ischemia (36 min).

### Biological rationale for impaired ATP recovery

**Figure 8:**
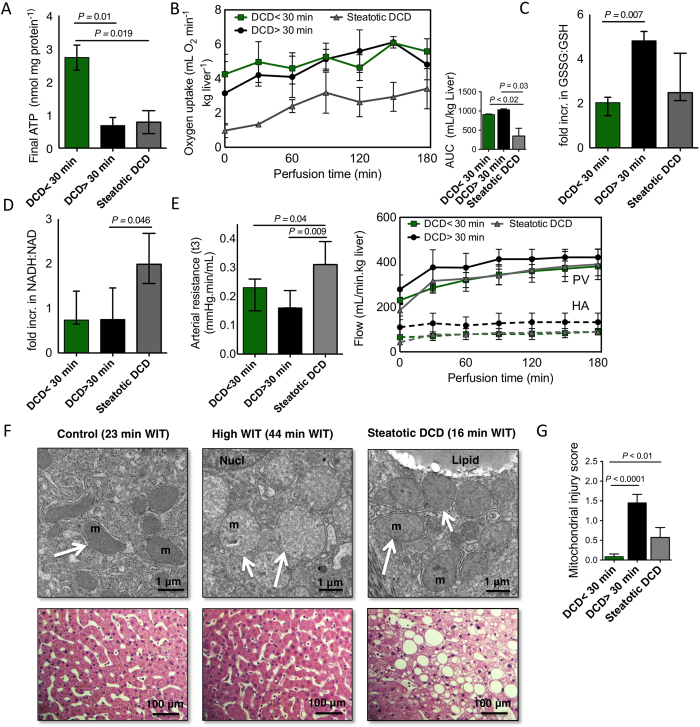
Mitochondrial respiratory function and ATP recovery. (**A**) Absolute ATP content at the end of perfusion in the different groups (n = 3). Final ATP levels were significantly lower in the DCD >30 min and steatotic DCD groups (*P *<* *0.02). (**B**) Oxygen uptake rates between groups throughout perfusion and total oxygen consumption, determined by the area under the curve. Total oxygen uptake was significantly lower in steatotic DCD livers, compared to the non-steatotic livers (DCD >30 min and DCD <30 min) (*P* < 0.03). (**C**) Change in ratio of oxidized to reduced glutathione (GSSG:GSH) during perfusion (t3/t0) in the different groups as an indicator of oxidative stress. DCD >30 min livers exhibited the largest increase in GSSG:GSH, with a significantly higher ratio change than the DCD <30 min group (*P *=* *0.007). (**D**) Change in ratio of NADH to NAD^+^ during perfusion in different groups. DCD <30 min and DCD >30 min groups show minimal change in NADH:NAD, while steatotic DCD livers exhibit a significant increase (*P* = 0.046). (**E**) Arterial resistance at the end of perfusion and flow through the portal vein (PV) and hepatic artery (HA). Arterial resistance was significantly higher in the steatotic DCD livers (*P* < 0.05), but the difference in flow through the arterial or portal system did not reach statistical significance (shown as mean ± sem). (**F**) Structural and ultrastructural preservation. Transmission electron microscopy images of representative DCD <30 min, DCD >30 min, and steatotic DCD livers (top). Corresponding H&E photomicrographs are shown in the bottom row. m, mitochondria; Nucl, nucleus; lipid; intracellular lipid droplet. (**G**) Mitochondrial injury scoring. Injury is scored based on the appearance of cristae, swelling and intramitochondrial deposition of amorphous material. Mitochondrial injury was increased in both DCD >30 min livers and steatotic DCD livers compared to DCD <30 min livers (n = 2 × 20 cells, mean ± sem) (*P *<* *0.01).

The reconstitution of ATP in the DCD >30 min group and steatotic DCD groups differed substantially from the controls (P < 0.02; Fig. 8A), reflecting compromised energy recovery during SNMP in these groups. Despite the poor recovery of ATP in the DCD >30 min group, oxygen uptake was comparable to the DCD <30 min group (Fig. 8B). Compared to the DCD <30 min, oxidation of glutathione (as fold change in GSSG:GSH) was found to be increased substantially in DCD >30 min livers (*P *=* *0.007; Fig. 8C). Similarly, the availability of NADPH was lower in this group (Fig. S1C).

Steatotic DCD livers showed significantly lower oxygen uptake than both DCD <30 min and DCD >30 min (*P* < 0.03; Fig. 8B). Oxidation of both glutathione and NADP^+^ was less in steatotic livers than in DCD >30 min (Figs 8C and S1C), while the ratio NADH:NAD^+^ increased significantly more in the steatotic group (*P* = 0.046; Fig. 8D). Steatotic DCD livers appeared to be more resistant to arterial flow, with a higher resistance at the end of perfusion (*P *=* *0.04), although flow rates were not significantly affected (Fig. 8E).

Transmission electron microscopy (TEM) revealed marked differences in mitochondrial ultrastructure between livers both before (not shown) and after perfusion, with significantly more injury in the DCD >30 min group (*P *<* *0.0001) and to a lesser extent in steatotic DCD livers (*P *=* *0.01; Fig. 8F (top) and G), which concurred with poor ATP recovery in these groups (Fig. 8A). After SNMP, mitochondria showed minimal swelling and retained discernable inner and outer membranes and cristae in the DCD <30 min group. Mitochondria of DCD >30 min livers were enlarged and electron-lucent, consistent with mitochondrial swelling as a result of increased membrane permeability. Steatotic DCD liver mitochondria showed a more heterogeneous appearance. While the majority of the mitochondria had a normal appearance, focal condensation was observed, characterized by increased mitochondrial electron density and dilation between the cristae. Representative H&E-stained histology sections are shown in Fig. 8F (bottom).

## Discussion

Nearly 40% of liver transplant candidates wait over 2 years for a liver transplant, while 10–15% of patients dies as a direct result of the insufficient supply of transplantable donor livers^20^,^21^. To increase this supply, the transplantation of DCD livers has increased substantially over the last 15 years, but appears to have plateaued at around 6% of transplanted livers^22^. Concerns for inferior long-term outcomes and biliary complications lead to a high discard rate of nearly 1 in 3 DCD livers^22^.

In recent years, *ex vivo* machine perfusion has shown convincing potential in increasing the viability of human livers and improving post-transplant outcome^7^,^23^. It was hypothesized that, by restoring oxygenation and providing metabolic substrates, machine perfusion allows for correction of metabolic perturbations inflicted during ischemia^24^. The understanding of these metabolic changes was mostly superficial, limiting not only the optimization of current *ex vivo* machine perfusion protocols, but importantly, also limiting the pretransplant assessment of hepatic function. Here, we present a comprehensive characterization of the *ex vivo* hepatic metabolic condition to provide insight into the processes that may contribute to recovery during SNMP. Moreover, by studying livers subjected to varying degrees of warm ischemic and steatotic injury, we aimed to improve the understanding of these injurious conditions and their *ex vivo* recovery.

The SNMP protocol employed here is based on the hypothesis that a controlled pretransplant period of SNMP supports hepatic function and allows for metabolic recovery of the liver prior to implantation. Moreover, we propose that metabolic recovery of the liver in the absence of leukocytes reduces reoxygenation-mediated injury, as evidenced by the mild degree of injury sustained during SNMP.

There is growing evidence showing that hepatic ATP content before transplantation and recovery of the energy status during reperfusion is positively correlated with favorable posttransplant outcome^4^,^25^,^26^. In clinical studies, a correlation between postpreservation^27^ or postreperfusion^28^,^29^,^30^ ATP levels and primary liver graft function was suggested. The metabolomic comparison between the ischemic and the perfused liver in this study shows a considerable recovery of energy metabolism during SNMP. Most notable were an increase in ATP and other adenine nucleotide ratios (such as energy status, which have been suggested to better represent energy status^31^). Additionally, NADPH:NADP^+^ ratios surpassed fresh levels after perfusion. NADPH increase is likely to result from the pentose phosphate pathway activity, supported by reduced Glu-6-P, in an attempt to fuel anabolic processes. Moreover, a redox shift towards NADPH would allow for improved oxygen radical scavenging capacity through an increased potential to reduce glutathione. Untargeted metabolomic analysis of the liver during perfusion most notably revealed a decrease in carbohydrate metabolites and an increase in amino acids and peptides. The observed increase in TCA cycle intermediates likely results from multiple anaplerotic reactions, including lactate-pyruvate conversion, but feasibly also from the catabolism of amino acids from the perfusion solution.

ATP depletion during normothermic ischemia results in a compensatory accelerated glycolysis and lactate accumulation^32^. The result on glycolytic intermediates is an increase in glucose-6-phosphate, fructose-6-phosphate, and a decrease in intermediates downstream of fructose-2,6-biphosphate^33^. When oxygenation is restored, a reversal is expected as observed here during SNMP. Urea production, which increased during SNMP, attests to an active amino acid catabolism during perfusion. Levels of amino acid were maintained and even increased, speculatively by a continuous supply in the perfusion media. The significance of an increased TCA intermediate pool can be functionally explained by the increased demand for electron transport chain (ETC) substrates following ischemia^34^.

During SNMP we observed a high variation in bile production between the 21 perfused livers. Moreover, severely ischemic and steatotic DCD groups had a significantly higher release of injury markers. This supported our hypothesis that metabolic assessment could discriminate different degrees of liver injury. Numerous clinical studies have shown an increased risk of graft failure in DCD livers^1^,^35^, with increasing WIT as an additional risk factor^36^. Similarly, macrovesicular steatosis of >30% is associated with a 60% higher risk of graft loss in the first posttransplant year^37^. In both cases, increased I/R injury is implicated, but different mechanisms are assumed to be responsible^38^. Under normal conditions most of the consumed oxygen is used for ATP production^39^, while only minimal amounts of consumed oxygen result in the production of O_2_^.−^ and derivative reactive transients and subsequent oxidative injury, which is reflected in the redox state of glutathione^40^. Healthy livers exhibit a high reconstitution of ATP and low electron leakage from the ETC, but in the case of prolonged ischemia mitochondrial dysfunction results in poor ATP production and increased formation of O_2_^.−^ during reperfusion^41^,^42^.

Building on this, the combination of perfusion parameters, metabolomics, and cofactor data gives us an indication of various mechanisms of injury and recovery and differences between groups. However, it should be noted that some of the data pertaining to energy metabolism are incongruent and currently difficult to fully consolidate mechanistically. Nonetheless, several hypotheses are supported. Livers subjected to <30 min WIT exhibited a similar oxygen extraction rate and fold increase in the NADH:NAD^+^ ratio compared with livers subjected to >30 min WIT, yet had higher hepatocellular ATP content than the livers with longer ischemia times. One could argue that the difference in ATP levels is attributable to the more abundant conversion of intracellular oxygen to superoxide (O_2_^.−^), as reflected in the increased GSSG:GSH ratio in the >30 min WIT group, which would reduce the available oxygen to act as terminal substrate for the ETC (at complex IV). After all, the magnitude of the GSSG:GSH ratio increase is approximately equal to the magnitude of the ATP decrease. This phenomenon does not have to affect the extent to which NADH is oxidized as long as the liberated electron can leave the ETC and react with oxygen, either leaking pre-complex IV (forming O_2_^.−^) or reacting with oxygen via complex IV (forming water). However, the notable mitochondrial structural aberrations in the >30 min WIT group compared to the relatively unaffected mitochondria in the WIT <30 min group strongly suggest that mitochondria are afflicted, which would most likely translate to perturbations in ETC function and the NADH:NAD^+^ ratio, favoring the accumulation of NADH. The observation that these ratios do not exhibit intergroup differences is therefore elusive. Moreover, the TCA cycle intermediates exhibit concentration differences between t0 and t3 with respect to the early intermediates (citrate and α-ketoglutarate), but these differences level out at the more downstream metabolites (succinate, fumarate, and malate). This tapered trend suggests that the catalytic rates, substrate affinities, or functionality of the enzymes that regulate the TCA cycle changed during SNMP, shifting the rate-limiting step from oxalosuccinate (before α-ketoglutarate) to succinyl-CoA or succinate. The exact cause for this process is unclear.

Interestingly, we found convincing indications of a distinctive mechanism at play in steatotic livers, which similarly demonstrated a poor recovery of ATP – while oxidative stress appeared more mitigated. Steatotic livers exhibit a compound sensitivity to I/R injury, perhaps secondary to a preexistent dysfunctional mitochondrial respiration^43^. Lipid accumulation increases hepatocyte size substantially and can decrease sinusoidal space by 50%^44^. The mechanical compression causes perturbed microcirculatory function that in turn results in compromised flow^45^. It has further been speculated that increased diffusional distances, in enlarged hepatocytes result in poor oxygen delivery^46^. We observed an increased arterial resistance in steatotic human livers during SNMP, however, flow was not sufficiently compromised to account for reduced oxygen consumption found in these grafts, suggesting a role of poor delivery of oxygen to the mitochondria. We show here that *ex vivo* machine perfusion can identify differences in mitochondrial respiration and recovery of energy stores between different groups of livers pretransplantation.

This study is limited by the lack of clinical transplantation or *ex vivo* simulated (pseudo-) reperfusion with whole blood to correlate our findings to a posttransplant or postreperfusion outcome. Nevertheless, the criteria with which livers were selected for metabolomic analysis allow cross-reference to posttransplant outcomes in large cohort studies. For instance, data from the United States Organ Procurement and Transplantation Network revealed that warm ischemic time (defined as in this study) in excess of 30 minutes increased the risk of graft loss by 77%, compared to low ischemic times (<15 min)^36^. Furthermore, owing to the heterogeneity of discarded livers, results in perfusion parameters (bile flow, oxygen uptake) and energy metabolism were highly variable between organs. Some of these parameters appeared in other work to be dependent on the donor characteristics used to identify subgroups here^6^. To avoid substantial heterogeneity in the subgroup analysis, metabolomic analysis and comparison between groups was limited to livers that met select inclusion criteria. As a result, a smaller number of livers could be included.

Although metabolomic analysis has seen considerable application in the study of hepatobiliary disease, it’s application in human liver transplantation remains relatively underexplored. In a recent study, the metabolome of pretransplant tissue biopsies was able to separate livers resulting in early allograft dysfunction, identifying potential 93 biomarkers associated with poor posttransplant function^11^. SNMP may improve on this cross-sectional metabolomics, allowing a dynamic evaluation of hepatic function. Dynamic metabolomic analyses have been performed to elucidate early posttransplant metabolic changes in both bile samples^13^ and sequential liver biopsies^12^. The clinical application of metabolomics would require rapid assessment of the donor liver to satisfy the time-constrained logistics of transplantation. In the near future, emerging techniques, such as rapid evaporative ionization mass spectrometry, which has shown use for *in situ* applications^47^, will enable near–real-time metabolomic profiling. This could help to distinguish organs that are transplantable from those that should be discarded. To identify definitive biomarkers during *ex vivo* assessment of the liver prospective testing is required with correlation to clinical outcome parameters, as these become available in the translation of machine perfusion to clinical application.

In conclusion, this comprehensive assessment of the liver during subnormothermic machine perfusion has revealed various metabolic changes that occur during functional preservation. Improvement of energetic cofactors and redox shifts during machine perfusion are likely to contribute to improved function after reperfusion. Untargeted metabolomic profiling was able to cluster livers with similar metabolic patterns based on the degrees of injury, including steatosis and severe warm ischemia in DCD livers. Moreover, perfusion parameters combined with differences in metabolic factors between steatotic and severely warm ischemic DCD livers suggested variable mechanisms that result in poor recovery of ATP in these livers. These results show great promise for the use of metabolomic analysis during machine perfusion of the liver. As new machine perfusion strategies are translated into clinical application, a tandem application with metabolomic assessment is warranted.

## Additional Information

**How to cite this article**: Bruinsma, B. G. *et al.* Metabolic profiling during *ex vivo* machine perfusion of the human liver. *Sci. Rep.* **6**, 22415; doi: 10.1038/srep22415 (2016).

## Supplementary Material

Supplementary Information

## Figures and Tables

**Figure 1:**
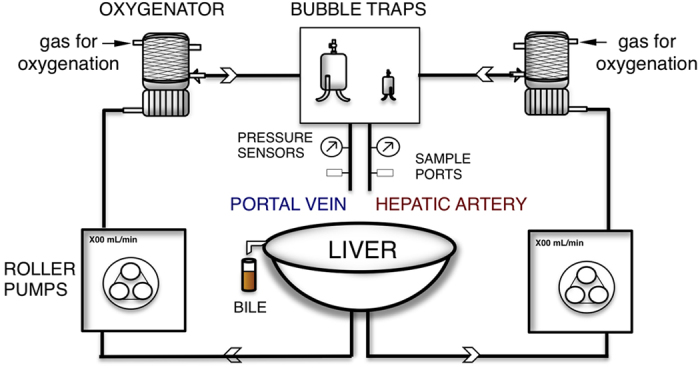
Schematic representation of the SNMP system. Two parallel circulations provide continuous flow of oxygenated perfusate to the liver through the portal vein (left) and hepatic artery (right). Flow and pressure on the vessels are monitored continuously. Oxygenation of the perfusate was provided by membrane oxygenators using carbogen gas(95% O_2_/5% CO_2_) mixture. The temperature is ambiently maintained at 21 ± 0.3 °C. The pressure of the system is controlled by flow adjustments to achieve target pressures of 3–7 mmHg and 30–80 mmHg on the PV and HA, respectively.

**Table 1:** Metabolite changes during SNMP.

Metabolite	Tissue metabolite levels(relative peak height x1000)		*P*value	AdjustedP value	Superclass	Class	Pathways
	*t0*	*t3*					
	Mean ± SD	Mean ± SD					
glycerol	34.37 ± 20.30	14.1 ± 4.08	*0.010*	0.086	Carbohydrates	Sugar alcohols	
xylitol	2.62 ± 2.42	0.37 ± 0.20	*0.013*	0.099			
ribulose-5-phosphate	0.31 ± 0.19	0.16 ± 0.08	*0.046*	0.233		Sugars (mono-, di-, tri)	Pentose Phosphate Pathway
N-acetylmannosamine	1.58 ± 0.84	0.60 ± 0.29	*0.005*	0.072			Sugar catabolism
maltose	68.96 ± 38.26	23.90 ± 16.89	*0.005*	0.072			Sugar catabolism
lactobionic acid	256.33 ± 171.19	126.1 ± 83.23	0.057	0.264			
hexose-6-phosphate	0.20 ± 0.14	0.11 ± 0.06	0.090	0.327			
glycerol-alpha-phosphate	108.7 ± 47.67	69.76 ± 35.40	0.067	0.280			
glucose-6-phosphate	0.58 ± 0.61	0.09 ± 0.04	*0.029*	0.161			Gluconeogenesis, Pentose Phosphate Pathway
fructose-6-phosphate	0.38 ± 0.28	0.13 ± 0.03	*0.015*	0.109			Gluconeogenesis, Pentosie Phophate Pathway
raffinose	46.51 ± 24.07	24.26 ± 16.38	*0.036*	0.188			Sugar catabolism
glyceric acid	0.45 ± 0.11	1.83 ± 1.55	*0.017*	0.116		Sugar Acids	
valine	23.1 ± 6.62	40.9 ± 9.02	*0.0002*	*0.012*	Amino acids and peptides	Amino Acids and Derivatives	Amino acid degredation, ammonia recycling
threonine	8.05 ± 3.58	16.5 ± 5.33	*0.001*	*0.032*			
serine	12.2 ± 5.08	22.5 ± 7.57	*0.004*	0.072			
proline	25.8 ± 9.67	42.95 ± 14.14	*0.008*	0.086			Amino acid degredation
N-acetylglutamate	0.27 ± 0.11	0.35 ± 0.28	0.074	0.294			
leucine	27.5 ± 4.62	48.93 ± 12.47	*0.0002*	*0.012*			Amino acid degredation
isoleucine	12.2 ± 3.74	28.0 ± 8.75	*0.0001*	*0.012*			Amino acid degredation
glutamine	12.2 ± 6.13	27.90 ± 17.88	*0.024*	0.158			Amino acid degredation, ammonia recycling, urea cycle
succinic acid	1.06 ± 0.86	2.15 ± 1.31	0.053	0.259	Organic acids and derivatives	Carboxylic Acids and Derivatives	TCA cycle
citric acid	0.81 ± 0.42	3.99 ± 3.24	*0.010*	0.086			TCA cycle
fumaric acid	9.05 ± 2.58	11.7 ± 3.48	0.088	0.327			TCA cycle, Urea cycle, amino acid degradation
alpha-ketoglutarate	0.09 ± 0.02	0.33 ± 0.24	*0.008*	0.086		Keto-Acids and Derivatives	TCA cycle, ammonia Recycling
lactic acid	131.5 ± 52.72	51.93 ± 27.61	*0.001*	*0.032*		Hydroxy Acids and Derivatives	Gluconeogenesis, Pyruvate metabolism
2-hydroxyglutaric acid	0.13 ± 0.03	0.21 ± 0.10	*0.030*	0.161			
UDP GlcNAc*	0.21 ± 0.11	0.13 ± 0.07	0.074	0.294	Nucleosides,	Pyrimidine Nucleotides	
guanosine	0.25 ± 0.09	0.50 ± 0.34	0.054	0.259	nucleotides, and analogues	Purine Nucleosides and Analogues	
urea	16.47 ± 13.34	34.1 ± 9.80	*0.006*	0.072	Miscelleneous	Ureas	Urea cycle, amino acid degredation
uric acid	2.43 ± 1.62	1.04 ± 0.60	*0.028*	0.161		Imidazopyrimidines	
phenylethylamine	0.54 ± 0.68	1.59 ± 1.50	0.077	0.298		Phenethylamines	
oxoproline	68.34 ± 32.34	119.8 ± 41.69	*0.010*	0.086		Pyrrolidines	Glutathione metabolism
N-acetyl-D-hexosamine	0.88 ± 0.41	0.35 ± 0.28	*0.005*	0.072		Unclassified	
lactamide	0.22 ± 0.12	0.14 ± 0.03	0.066	0.280		Unclassified	

*Uridine diphosphate-N-acetylglucosamine.
