# Supplementary material for: Metabolic profiling during ex vivo machine perfusion of the human liver
Source: Sci Rep. 2016 Mar 3;6:22415. doi: 10.1038/srep22415 (PMC4776101; doi:10.1038/srep22415)
Supplement: Supplementary Information [file srep22415-s1.pdf]

SUPPLEMENTARY MATERIALS

Metabolic profiling during ex vivo machine perfusion of the human liver

Bote G. Bruinsma[1,2], Gautham V. Sridharan[1], Pepijn D. Weeder[1,3], James H. Avruch[4], Michal Heger[2], Nima Saeidi[1], Sinan Özer[1], Sharon Geerts[1], Robert J. Porte[1], Michal Heger[2], Thomas M. van Gulik[2], Paulo N. Martins[5], James F. Markmann[4], Heidi Yeh[4], Korkut Uygun[1]*

*corresponding author

korkut.uygun@mgh.harvard.edu

[1]Center for Engineering in Medicine, Dept. of Surgery, Massachusetts General Hospital, Harvard Medical School, Boston, MA, USA

[2]Department of Experimental Surgery, Academic Medical Center, University of Amsterdam, Amsterdam, The Netherlands

[3] Section of Hepatobiliary Surgery and Liver Transplantation, Department of Surgery, University Medical Center Groningen, University of Groningen, Groningen, The Netherlands

[4]Transplant Center, Dept. of Surgery, Massachusetts General Hospital, Boston, MA, USA

[5] Transplant Division, Dept. of Surgery, University of Massachusetts, Worcester, MA, USA

SUPPLEMENTARY METHODS

Principal Component Analysis (PCA). The metabolomics data for the initial and final biopsies were projected onto two principal components to enable visualization of the overall metabolic shift between pre and post perfusion for the 9 livers. The t=0 h time point and the t=3 h time point biopsies were treated as independent, resulting in 18 observations. An 18X159 matrix N was then constructed with rows as observations and columns for measured metabolites, where every entry N(i,j) was computed as the median peak height for the three triplicate samples. Principal components and 95% confidence band ellipses were computed using Matlab (Mathworks, Natick, MA), using z-scores of each matrix entry relative to other entries in the same column, which prevented metabolites with significantly higher average peak heights from dominating relative contribution towards the principal components.

Heatmap. The metabolomics data are were organized as two 159X9 matrices for each time point, where the rows were denoted by measured metabolites and the columns by each liver, and each entry is the calculated median of the peak intensities from three technical replicates of the same biopsy. Since the peak intensity values range several orders of magnitude, the values in each matrix were replaced by their z-score with respect to the nine entries in each row. In both the pre and post-perfusion data matrices, the minimum calculated z-score was -2.05 and the maximum z-score was 2.65. In this regard, a higher z-score represents a relatively higher abundance of that metabolite in the specific liver compared to other livers at the same time point.

The z-scores were then presented as two heatmaps with metabolite rows and livers in columns with the first heatmap comparing the control livers to warm ischemic livers (Fig 5A) and the second comparing control livers to the steatotic group (Fig 5B) at pre-

perfusion. The color gradient used to color the entries ranges from green to red, corresponding to relatively low and high abundance of the metabolite respectively. The order of the metabolite rows is determined by the slope of the least-squares line fit through a scatter of z-score versus WIT or z-score versus degree of steatosis (which was just assigned a binary for 0 for no steatosis and 1 for steatosis) in increasing order. For each row, we also compute the correlation coefficient of that scatter and a *P*-value of whether or not that correlation is significant using the Matlab function corrcoef, which reports the probability of obtaining a correlation as large as reported by random chance when the true correlation is zero. Metabolites for which the correlation *P*-value was ≤ 0.05 were deemed as significantly correlated to either WIT or steatosis, metabolites with for which the correlation *P*-value ≤0.10 are presented in Table S2.

Targeted metabolomics – cofactor analysis. Crushed tissue biopsies (averaging ~25 mg) were also analyzed for metabolic cofactors using a targeted MRM (multiple reaction monitoring) analysis on a 3200 QTRAP LC/MS-MS (Triple quadrupole liquid chromatography – mass spectrometry) system (AB Sciex, Foster City, CA). The metabolites were first extracted using 250 µL of a 2/1 (v/v) mixture of methanol/chloroform and were subject to three freeze-thaw cycles, which entailed 30 seconds of rapid freezing in liquid nitrogen, thawing at room temperature, and a 10 second vortex. Ice cold water (200 µL) was then added to each extract and after a 1-minute centrifugation at 15,000 g, the upper phase of the resulting biphasic mixture was transferred to an HPLC autosampler vial for LC/MS analysis. The chromatographic separation conditions and the analyte-specific MS parameter optimization routines were the same as that reported in Quinn et al., for which NAD^+, NADH, and FAD were quantified(1). In this study, MRM transitions for ATP, ADP, AMP, NADPH, NADP, GSH, and GSSG were also quantified in addition to NAD^+, NADH, and FAD. The precursor/product ion transitions for all the compounds are

presented in Table S3. The peak area of each MRM transition was correlated to extract concentration based on serial dilutions of pure chemical standards. Pertinent redox ratios were then computed based on the relative concentrations of cofactors calculated to be in the tissue extract.

Untargeted metabolomics analysis. An untargeted profiling of primary metabolites was performed at the West Coast Metabolomics Center (Davis, CA) using GC-TOF-MS (gas chromatography – time of flight – mass spectrometry). Briefly, the homogenized biopsies were subject to solvent-based extraction using a mixture of acetonitrile, isopropanol and water as described in Fiehn et al. (2). This was followed by a clean-up procedure using acetonitrile/water to remove triglycerides and lipids, after which internal standards were then spiked to the extracted sample. The details of the mass spectrometry data acquisition, including the chromatography conditions and MS operating parameters, have been outlined elsewhere(2). The raw data were provided for each identified metabolite as peak heights normalized to the total ion chromatogram (sum of all peaks) to account for variation in tissue mass and total quantity of extracted metabolites. Peak heights were deemed more precise and reproducible than peak areas for quantification, particularly for low-abundance metabolites. The final raw data were reported as a 159X54 matrix, organized by the 159 detected metabolites for 54 crushed biopsy samples sent for metabolomics analysis from the 9 livers at 2 time points (t=0 h and t=3 h), each with 3 replicates.

The Benjamini & Hochberg and the Benjamini Yekutieli procedure for controlling the false discovery rate (FDR) of a family of hypothesis tests were used to correct for multiple comparisons.

SUPPLEMENTARY REFERENCES

1) Quinn, K. P. *et al.* Quantitative metabolic imaging using endogenous fluorescence to detect stem cell differentiation. *Sci Rep* **3,** 3432 (2013).

2) Fiehn, O. *et al.* Quality control for plant metabolomics: reporting MSI-compliant studies. *Plant J* **53,** 691-704 (2008).

SUPPLEMENTARY FIGURE 1

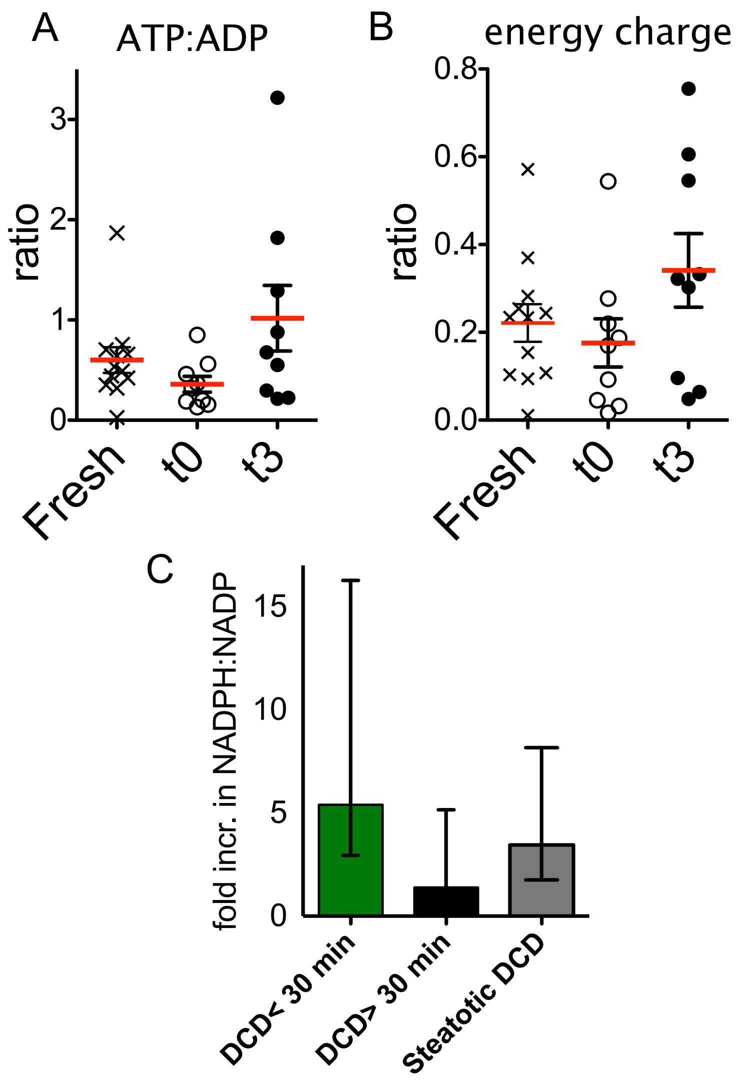

Fig. S1. Adenine and nicotinamide cofactor ratios. (A) Changes in adenosine triphosphate (ATP) to adenosine diphophate (ADP) and **(B)** energy charge during SNMP. Energy charge is calculated as (ATP + 1/2 ADP)/(ATP+ADP+AMP). **(C)** Change in ratio of NADP:NADPH during perfusion in different liver groups (n=3). Bars in A/B represent mean ± sem.

SUPPLEMENTARY TABLES

TABLE S1 – Donor liver characteristics

		Donor type	Gender	Age	COD	CIT	relative WIT	peak AST	peak ALT	final AST	final ALT	Macrosteatosis score	Microsteatosis score	ICG
DCD< 30 min	1	DCD	M	54	Stroke	559	19	N/A	N/A	N/A	N/A	0	0	
	2	DCD	F	42	Anoxia	432	19	664	145	255	257	0	0	*
	3	DCD	M	46	Head Trauma	416	23	29	15	35	15	0	0	
DCD> 30 min	4	DCD	M	35	Head Trauma	447	36	183	162	183	162	0	0	*
	5	DCD	F	66	Stroke	377	44	32	12	30	12	0	0	
	6	DCD	M	50	Anoxia	1157	54	103	118	103	118	0	0	
Steatotic DCD	7	DCD	M	44	Anoxia	789	16	86	61	60	38	3	2	
	8	DCD	F	69	Head trauma	342	24	621	465	621	395	3	2	*
	9	DCD	F	68	Stroke	332	27	201	60	201	60	2	3	
	10	DCD	M	25	Anoxia	1002	23	N/A	N/A	N/A	N/A	1	1	*
	11	DCD	M	50	Head trauma	296	28	57	42	41	23	1	1	
	12	DCD	F	62	Resp. failure	740	34	21	8	19	5	1	1	
	13	DBD	M	75	Head trauma	685	N/A	47	31	35	24	2	2	*
	14	DBD	M	24	Anoxia	420	N/A	485	374	39	155	4	4	
	15	DCD	M	51	Anoxia	525	20	608	288	82	62	1	1	
	16	DCD	M	63	Anoxia	737	28	N/A	N/A	N/A	N/A	1	0	
	17	DCD	M	57	Head trauma	250	21	230	144	230	115	2	2	*
	18	DBD	M	65	Anoxia	360	N/A	801	489	273	224	4	4	
	19	DCD	M	21	Head Trauma	420	14	81	14	81	14	1	1	*
	20	DCD	N/A	49	N/A	431	21	N/A	N/A	N/A	N/A	0	2	
	21	DCD	M	21	Anoxia	450	40	77	37	41	29	1	0	

** indocyanine green clearance*
Grey shaded livers were included in the metabolomic analysis. Livers 1-3, 4-6, and 7-9 were included in the DCD< 30 min, DCD> 30 min, and steatotic DCD group respectively.

TABLE S2 – Discriminating metabolites

DCD< 30 min vs. DCD> 30 min t0

Metabolite	Correlation	*P* value
2-ketoisocaproic acid	0.76	0.08
arachidic acid	0.86	0.03
asparagine	0.75	0.09
benzoic acid	0.74	0.09
cholestan-3-ol	0.85	0.03
cholesterol	0.78	0.07
creatinine	0.79	0.06
cysteine	0.83	0.04
cysteine-glycine	0.82	0.04
cystine	0.82	0.05
dehydroabietic acid	0.80	0.06
dodecanoic acid, isopropan	0.76	0.08
epsilon-caprolactam	0.96	0.00
glutaric acid	0.89	0.02
glycolic acid	0.85	0.03
guanosine	0.76	0.08
heptadecanoic acid	0.76	0.08
lactobionic acid	-0.77	0.07
lauric acid	0.84	0.03
linoleic acid	0.85	0.03
maltose	-0.79	0.06
maltotriose	0.77	0.07
ornithine	0.75	0.08
palmitic acid	0.73	0.10
pelargonic acid	0.74	0.09
proline	0.92	0.01
pyrophosphate	-0.79	0.06
raffinose	-0.76	0.08
stearic acid	0.81	0.05
tocopherol gamma-	0.89	0.02
tyrosine	-0.80	0.06
uridine-5-monophosphate	-0.81	0.05
xylitol	0.73	0.10

DCD< 30 min vs. DCD> 30 min t3

Metabolite	Correlation	*P* value
2-hydroxybutanoic acid	-0.76	0.08
cholesterol	0.86	0.03
cystine	0.93	0.01
glyceric acid	0.84	0.04
glycerol-alpha-phosphate	-0.79	0.06
inosine	0.88	0.02
inosine-5'-monophosphate	-0.89	0.02
inositol-4-monophosphate	0.83	0.04
lactobionic acid	-0.78	0.07
lysine	0.74	0.1
methanolphosphate	0.74	0.1
serine	0.86	0.03
tocopherol gamma-	0.83	0.04
uric acid	0.85	0.03
uridine-5-monophosphate	-0.75	0.08
xanthine	0.86	0.03
xanthosine	0.75	0.09

DCD< 30 min vs. Steatotic DCD t0

Metabolite	Correlation	*P* value
cellobiose	-0.73	0.1
citric acid	-0.99	<0.005
dodecanol	-0.74	0.09
ethanolamine	-0.91	0.01
fructose	0.92	0.01
glutaric acid	0.82	0.05
glyceric acid	0.75	0.08
histidine	-0.77	0.07
hypoxanthine	-0.77	0.07
inosine-5'-monophosphate	-0.79	0.06
inositol-4-monophosphate	-0.73	0.1
levoglucosan	-0.81	0.05
linoleic acid	0.77	0.08
mannose	0.87	0.02
methanolphosphate	-0.82	0.04
nicotinamide	-0.89	0.02
octadecanol	-0.75	0.09
p-cresol	-0.74	0.09
phosphate	-0.74	0.09
phosphoethanolamine	-0.79	0.06
pipecolinic acid	-0.74	0.1
ribose-5-phosphate	-0.74	0.09
squalene	-0.94	0.01
tagatose	0.93	0.01
tyrosine	-0.79	0.06
uracil	0.94	<0.005
urea	0.76	0.08
uridine-5-monophosphate	-0.95	<0.005
xylulose NIST	0.84	0.04

DCD< 30 min vs. Steatotic DCD t3

Metabolite	Correlation	*P* value
2-hydroxybutanoic acid	-0.74	0.09
3-phosphoglycerate	0.85	0.03
adenine	-0.73	0.1
adenosine-5-monophosphate	-0.83	0.04
ascorbic acid	-0.76	0.08
asparagine	-0.87	0.02
beta-alanine	0.74	0.09
dehydroascorbic acid	-0.93	0.01
fructose	0.75	0.09
glucose	0.8	0.05
glucose-1-phosphate	-0.77	0.07
glutamine	-0.74	0.09
glyceric acid	0.74	0.1
guanosine	0.79	0.06
histidine	-0.85	0.03
hydroxylamine	0.82	0.05
inosine	0.83	0.04
inosine-5'-monophosphate	-0.97	<0.005
inositol-4-monophosphate	0.89	0.02
lactic acid	0.74	0.09
linoleic acid	0.93	0.01
lyxitol	0.89	0.02
malic acid	0.75	0.09
maltotriose	0.75	0.09
mannose	0.78	0.07
N-acetyl-D-hexosamine	0.93	0.01
N-acetylmannosamine	0.78	0.07
oleic acid	0.78	0.07
oxoproline	-0.84	0.04
pipecolinic acid	-0.75	0.09
pyrophosphate	-0.85	0.03
ribitol	0.89	0.02
ribose-5-phosphate	-0.76	0.08
threonine	-0.96	<0.005
UDP-N-acetylglucosamine	-0.86	0.03
uric acid	0.76	0.08
xanthosine	0.8	0.05
xylitol	0.94	<0.005

TABLE S3 – precursor/product ion transitions for targeted metabolomic analysis

ID	Q1 Mas	Q3 Mas	Time	DP (volt)	EP (volt)	CE (volt)	CXP (volt)
ATP	505.945	78.900	100	-50.000	-5.000	-100.000	-6.000
ADP	425.972	78.900	100	-95.000	-1.500	-88.000	-10.000
NADP	742.116	620.000	100	-15.000	-3.500	-24.000	-18.000
NAD	662.213	540.200	100	-10.000	-10.500	-22.000	-22.000
NADH	664.146	78.900	100	-65.000	-4.500	-128.000	-4.000
NADPH	744.112	78.900	100	-65.000	-9.800	-118.000	-4.000
AMP	326.000	78.900	100	-40.000	-10.000	-56.000	-6.000
FAD	784.100	79.000	100	-55.000	-9.000	-130.000	-2.000
GSSG	611.100	306.000	100	-30.000	-3.000	-26.000	-2.000
GSH	503.600	142.800	100	-30.000	-4.000	-26.000	-2.000
